# Single-particle cryo-EM: beyond the resolution

**DOI:** 10.1093/nsr/nwz127

**Published:** 2019-09-02

**Authors:** Jean-Paul Armache, Yifan Cheng

**Affiliations:** 1 Department of Biochemistry and Molecular Biology and the Huck Institutes of the Life Sciences, Pennsylvania State University, University Park, PA 16802, USA; 2 Howard Hughes Medical Institute, University of California San Francisco, USA; 3 Department of Biochemistry and Biophysics, University of California San Francisco, USA

Structural biology aims to provide structural insights into our understandings of biological processes at the atomic level. Atomic structures of biological macromolecules are often determined by one of the three major methods: X-ray crystallography, NMR spectroscopy and cryo-electron microscopy (cryo-EM) or, more specifically, single-particle cryo-EM. While X-ray crystallography has contributed most of the atomic structures in the protein databank, recent technological breakthroughs in single-particle cryo-EM have changed landscape of structural biology in a dramatic and revolutionary manner [1]. Many challenging biological questions that were considered too difficult to be studied structurally even a few years ago have now been, or are being, studied by single-particle cryo-EM, providing novel insights into our understandings of many important biological processes at the molecular and atomic levels [2].

While NMR spectroscopy is widely used to study protein dynamics, a major goal of structure determination in X-ray crystallography had been to achieve high enough resolution sufficient for *de novo* atomic model building of the target macromolecules. That is also true for single-particle cryo-EM, where almost every project aims to determine structures of the target molecules at the highest possible resolution. Obviously and undeniably, a reconstruction at a high resolution is very exciting because it provides intricate structural insight into mechanistic questions. Many studies, such as those aiming to understand the details of small-molecule ligand-binding, would require such high-resolution information to reveal details concerning, for instance, side-chain ligand interactions to understand the detailed ligand-action mechanism. This has become feasible as a result of a major breakthrough in single-particle cryo-EM, the so-called ‘resolution revolution’ [3]. In the last several years, much of the technological-development efforts in single-particle cryo-EM have been focused on pushing the resolution limits further. Current technologies are capable of producing 3D reconstructions at resolutions close to or better than 2 Å rather routinely [4]. The benefits of achieving higher-resolution structures are obvious, including unambiguous modeling of small molecules bound to target macromolecules, which facilitates structure-based drug discovery and development using single-particle cryo-EM, etc.

When the overall resolution of a reconstruction fails to reach the desired level, either to resolve side chains or to visualize secondary structural features, one would first try to find out what limits the resolution. Being able to identify the source of the problem that limits the resolution often determines the fate of the project, resulting with either success or failure. The first things to check are the properties of the cryo-EM data, particularly the quality of micrographs and cryo-samples. The quality of the micrographs can be assessed quantitatively and rather straightforwardly by parameters such as visibility of Thon rings at high resolution, defocus values, astigmatism and image contrast. Nowadays, almost all contrast-transfer-function determination software provides estimation of resolution limits of each micrograph, in addition to determining the defocus and astigmatism of these micrographs. Other factors are related to sample preparation, including ice thickness, particle distribution, angular distribution of particles, etc. Detecting preferred orientation is also straightforward and estimation of its influence on resolution or map quality can also be quantitative, although resolving the problem is often not straightforward [5,6]. In some cases, the alignment accuracy of particle images is a concern. This is particularly so for very small and globular molecules. Adding a fiducial marker such as a fragment of antigen binding (Fab) or a nanobody with conformational epitopes is often the approach to consider [7].

Next, one would assess the biochemical properties of the sample—particularly its conformational and compositional homogeneity. Heterogeneity, either conformational or compositional, could be caused by biochemical-purification procedures or damage from exposing proteins to the air–water interface during the process of preparing a frozen hydrated cryo-EM grid. Cross-linking is a feasible approach to dealing with fragile complexes, although this is often considered as the last option if other methods fail to produce satisfactory results.

The situation could be more complicated or frustrating when one achieves an atomic-level overall resolution yet a part of the molecule is poorly resolved. This is rather common in many single-particle cryo-EM projects, ranging from integral-membrane proteins to large macromolecular machineries or assemblies. In such situations, the cause of such poorly resolved regions is then clearly related to the sample homogeneity/heterogeneity rather than quality of the electron micrographs or the performance of the electron microscope. If the unresolved part is of critical biological importance, one often spends a tremendous amount of time and effort to resolve it, mostly by optimizing the sample preparation, ranging from ortholog screening to buffer optimization. In some cases, optimizing the biochemical conditions of the protein purification may improve the situation.

**Figure 1:**
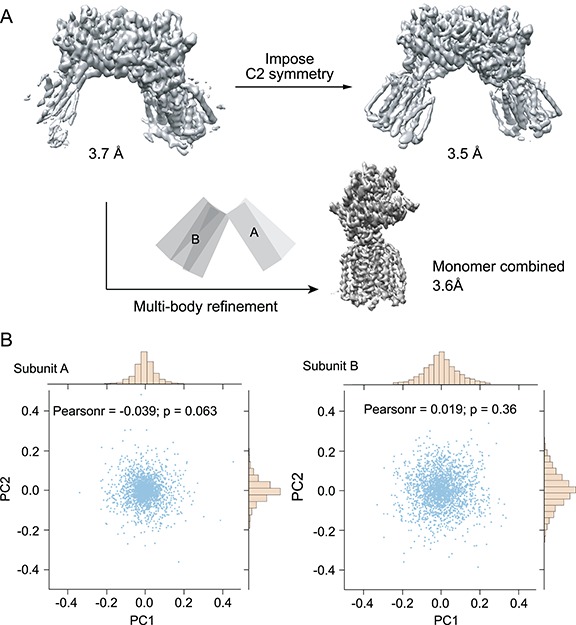
Flexibility of the Hedgehog Receptor Patched dimer. (A) The reconstruction of the Patched dimer shows that one of the monomers is better resolved, suggesting two subunits have motion with respect to one another. Multi-body refinement instead of C2 symmetrization improves the quality of the final map. (B) Motion of each monomer was characterized by kernel principle component analysis (kPCA) using geodesic kernel on the SO [3] manifold. The first two components for each subunit were plotted. This figure is adapted from [9].

For instance, a number of recently published ion-channel structures included cholesterol hemisuccinate (CHS) during protein purification [8]; this often leads to better-behaved proteins because the addition of CHS often stabilizes the membrane proteins. Efforts such as this may eventually lead to resolution improvements of an unresolved region. If conformational heterogeneity can be described as a rigid-body motion of individual domains, dividing a whole molecule into different domains and aligning them separately as rigid bodies could lead to improved resolution [9]. In cases like this, in addition to achieving high resolution, it is also important to characterize the relative motion of these ‘rigid’ domains, as shown in the example in Fig. 1.

A common and more brute-force approach to dealing with heterogeneity, either compositional, conformational or both, is simply collecting more micrographs and increasing the total number of particles in a dataset so that intensive 2D and/or 3D classifications would facilitate sorting out a sub-population of particles that are homogeneous enough to produce high-resolution structures. This approach could potentially lead to structures with better resolution. The drastically increased efficiency of data collection and power of computation allows the acquisition and processing of very large cryo-EM datasets with millions of particles. Sophisticated computational algorithms and complicated classification procedures can facilitate selecting a subset of particles of homogeneous conformation to reconstruct a high-resolution structure. An obvious but often ignored question related to such an approach is: what are those discarded particles? This question is, however, rather complicated or difficult to answer. One simple and straightforward (but somewhat hand-waving) answer is that those discarded particles are from molecules being damaged during sample preparation, particularly during cryo-grid preparation, during which the air–water interface is considered to be a primary source of damage to many particles, particularly fragile complexes.

Another possibility is that many of the discarded particles represent different conformations or states of the complexes. If this is the case, then the final high-resolution reconstruction obtained from a highly selected small subset of particles corresponds only to a specific conformation of the target complex among many other equally relevant conformations. Therefore, in such a case, it is necessary to think beyond the resolution and to seek out additional information on protein dynamics that may be equally important in function.

**Figure 2:**
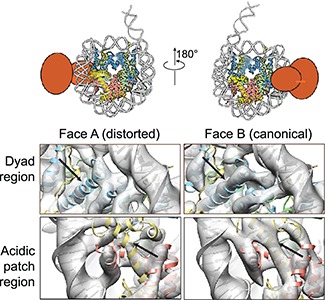
Missing histone densities are interpreted as histone mobility in nucleosome. A reconstruction of a nucleosome with a single SNF2h remodeler bound at the SHL+2 position reveals weaker density at the histone H2A acidic patch (bottom row) and the α2 helix of H3 (top row) in Face A when compared with the densities of the corresponding helices in Face B. This was interpreted as being caused by an asymmetrical binding of an ATP-dependent chromatin remodeler. This figure is adapted from [10].

An example of our own work concerns the structure of an ATP-dependent chromatin remodeler in a complex with a nucleosome [10]. Due to technical and historical reasons, one of the structures we determined was limited to sub-nanometer resolution (~7–8 Å) because the data were collected prior to the ‘resolution revolution’ using a scintillator-based camera. At this resolution, secondary-structure features are clearly resolved, including the α-helices of histones within the nucleosome. In this reconstruction, the densities of two helices in the histone were incomplete (Fig. 2). It is easy to interpret such missing densities as due to insufficient resolution, in which case the solution is to improve the quality of the map, either by changing buffer conditions or by increasing the size of the dataset so that more intensive classification would lead to a conformationally homogeneous subset. The counter-argument is that this is not related to insufficient resolution, but rather the intrinsic conformational heterogeneity arising from a specific functional feature of the nucleosome in this complex. The evidence supporting the intrinsic-heterogeneity interpretation is the fact that the densities of pseudo-symmetry-related helices are well resolved. If one pursues a high-resolution reconstruction in which all parts of the nucleosome are equally well resolved, one would ignore (or miss) the possibility that these two helices are functionally more dynamic. It is also worth emphasizing that interpreting a missing density as conformational dynamics or heterogeneity that is functionally related is not straightforward and would require additional validation, particularly biochemical data. In the case of the structure of the nucleosome–SNF2h complex, the latter was our interpretation and it was backed up by prior biochemical studies of the same complexes [11]. Similar observations of structural alternation in a histone octamer were also reported recently from another laboratory [12].

One of the next challenges for technological-development efforts in single-particle cryo-EM therefore might be focused on how to analyse such protein dynamics, how to evaluate and validate the result and how to relate them to protein function. The manifold approach developed in the past few years represents one of such efforts towards understanding conformational dynamics by using single-particle cryo-EM [13]. With increasing capabilities of computational resources, one can expect improvements and development of other methods in the future.
